# The restorative effect of platelet-rich plasma on estrous cycle disruption induced by arcuate nucleus lesion in female Wistar rats: An experimental study

**DOI:** 10.18502/ijrm.v23i1.18185

**Published:** 2025-03-21

**Authors:** Elham Abbasi, Morteza Behnam Rassouli, Ali Moghimi, Zeinab Neshati

**Affiliations:** Department of Biology, Faculty of Sciences, Ferdowsi University of Mashhad, Mashhad, Iran.

**Keywords:** Arcuate nucleus, Estrus cycle, PRP, Rat.

## Abstract

**Background:**

Successful reproduction relies on a functioning hypothalamic-pituitary-gonad axis. Damage to this axis disrupts the estrus cycle and reproductive capability.

**Objective:**

This study aimed to evaluate the effects of single or multiple platelet-rich plasma (PRP) injections on repairing the damaged hypothalamic arcuate nucleus (ARC) and restoring the estrus cycle in Wistar rats.

**Materials and Methods:**

90 female Wistar rats (2–3 months old, 250–280 gr) with regular estrous cycles were divided into a control group and 8 experimental groups (n = 10/each). After bilateral stereotaxic chemical surgery of the ARC using quinolinic acid (500 nmol/2 μ l), the experimental rats were categorized into several treatment regimens: ARC ${\;}^{-}$ (no treatment), 1 PRP injection (immediately, 24 hr, 48 hr, and 72 hr postsurgery), 2 PRP injections (immediately, 24 hr), 3 PRP injections (immediately, 24 hr, and 48 hr), and 4 PRP injections (immediately, 24 hr, 48 hr, and 72 hr). Vaginal smear cytology was performed daily for 2.5 months. In the end, rats brains were removed and divided for real-time polymerase chain reaction analysis of kisspeptin, neurokinin B, and dynorphin, as well as for ARC cell counting.

**Results:**

Vaginal smear cytology indicated that PRP administration gradually restored the estrous cycle. Compared to the ARC ${\;}^{-}$ group, PRP treatment significantly increased ARC cell density (p = 0.012) and mRNA levels of kisspeptin, neurokinin B, and dynorphin (p < 0.001).

**Conclusion:**

These findings not only emphasized the importance of the ARC for the regularity of estrous cycle but also showed the potential effects of local PRP treatment in contribution to the protection/reconstruction of ARC.

## 1. Introduction

In mammals, fertility begins with sexual maturation and pulsatile release of the gonadotropin-releasing hormone (GnRH) in the hypothalamus (1). GnRH, after being secreted in the median eminence region of the hypothalamus and transferred to the adenohypophysis through the hypothalamic-pituitary portal vessels, causes stimulation of gonadotrophs to release luteinizing hormone (LH) and follicle-stimulating hormone (2).

Although in males and females, after maturity and until the end of the reproductive period, GnRH secretion from GnRH neurons continues in a pulsatile manner. In females, in addition to pulsatile secretion, in the middle of each sexual cycle, a surge in GnRH secretion takes place (3). The surge of GnRH release, followed by an LH surge, is a chemical signal to the ovary to release ova. In rats, the pulsatile and surge release of GnRH is regulated by kisspeptin1 (kiss1) neurons located in the arcuate nucleus (ARC) and anteroventral periventricular nucleus of the hypothalamus, respectively (4, 5). ARC kiss1 neurons stimulate GnRH neurons by the release of kiss1 (6). There are also neurons that, apart from kiss1, release neurokinin B (NKB), dynorphin (Dyn), and kisspeptin neurokinin B dynorphin (KNDy) neurons. The latter plays a role in controlling GnRH pulses (7).

Both central and peripheral administration of kiss1 results in an elevation of LH levels in both humans and animals (8). Chemical destruction of the ARC causes the cessation of the estrous cycle. Similarly, male and female transgenic rats with a lack of the *kiss1* receptor gene (*kiss1r* or *Gpr54*) or lacking the *KISS1* gene do not develop sexual maturity and are infertile (9, 10). Genes encoding kiss1r/KISS1 are associated not only with congenital hypogonadism caused by gonadotropin hormone deficiency but also with maturation arrest (11). It was demonstrated that disrupting the rat ARC nucleus using stereotaxic methods and neurotoxin drugs led to disturbances in the estrous cycle (12).

Platelet-rich plasma (PRP) can be generally described as a volume of plasma with a high concentration of platelets. It has been found that platelets contain more than 1100 proteins (13). Among them are growth factors and regulators of the immune system, enzymes, and other biological compounds that play a role in various stages of the tissue repair processes. Recently, PRP application has been used to introduce a high concentration of factors and other biomolecules to damaged tissues (14, 15). In the case of nervous system damage, local administration of PRP can increase, in a dose-dependent manner, the myelination and regeneration of severed axons (16), and when combined with acellular nerve allografts leads to better electrophysiological and neurological outcome (17). Accelerating platelet activation and platelet gel formation by adding extra thrombin and calcium might facilitate the transportation of growth factors, contributing to nerve repair (18).

This research seeks to investigate the potential of PRP in repairing damage to the hypothalamic-pituitary-gonad axis and re-establishing normal reproductive function following injury to this axis.

## 2. Materials and Methods

### Laboratory animals

In this experimental study, 90 adult female Wistar rats (2–3 months old, 250–280 gr) were used. Animals were bred and utilized in the animal facility of the Faculty of Science, Ferdowsi University of Mashhad, Mashhad, Iran. The animals were kept in the animal room under standard conditions of 21–23 C, 12 hr light/dark cycle, with free access to food and water*, *ad libitum. At first, daily vaginal smear samples were taken from all rats to confirm the regularity of the estrus cycle. Then, they were randomly assigned into one control (intact) and 8 experimental groups (n = 10/each) (Table I). All experimental rats underwent bilateral stereotaxic chemical surgery of ARC (quinolinic acid; QA, 500 nmol/2 μ l, Merck, Germany) (19, 20).

### Method of preparing vaginal smears from rats

Daily vaginal smears from rats were prepared using swabs moisture with 0.9% NaCl solution and spread on a clean glass slide, stained with 1% methylene blue (Sigma, Germany), and then examined with a light microscope (Olympus Bx51, Japan). The cytologic criteria used for the recognition of estrus cycle phases were as follows; in the pro-estrus phase, the cells are the same shape and size as round nuclei; in the estrous phase, the cells are keratinized and polygonal without nuclei. A few keratinized cells with many leukocytes and multinucleated leukocytes with a few epithelial and keratinized cells are the cytologic appearance of met-estrus and di-estrus phases, respectively.

### Stereotaxic surgery

The rats underwent bilateral cannulation (in the ARC of the hypothalamus). First, each animal was anesthetized with a mixture of ketamine (100 mg/kg, Alphasen, the Netherlands) and xylazine (10 mg/kg, Alphasen, the Netherlands). During anesthesia and surgery, the animal's body was kept warm. For surgery, the animal's head was fixed on a stereotaxic device (Narishiii, Japan) and the animal's head hair was shaved from above the eyes to the bottom of the ears. The coordinates of the ARC nucleus using the Watson-Paxinus atlas were anteroposterior: -2.4 mm, mediolateral: ± 0.3 mm, and dorsoventral: 10 mm. After marking the 2 desired points on the right and left sides of the skull surface, the 2 marked points were drilled, and the cannulas were guided to the desired points, which were fixed using acrylate cement. Then, 500 nmol in 2 μ l of QA was injected into both sides of the brain with a Hamilton syringe through a very slow cannula over 5 min to destroy the ARC bilaterally.

### PRP preparation and injection 

Blood was collected from a normal rat heart to prepare PRP and poured into an ethylene-diaminetetra-acetic acid tube. The first centrifuge was set for 10 min with 2000 rounds/min, and the second centrifugation was set for 10 min with 4000 rounds/min. Then, blood plasma containing platelets was extracted using a micropipette, and diluted with normal saline. It was injected with a 1.72 × 10^6^ of platelet in 5 µL volume per rat into the lesioned ARC through the cannulas (21). After 3 wk of PRP injections, to check the estrous cycle status, daily vaginal smears were taken.

### Removing the brain for histological study

At the end of the experimental period (2.5 months), the animals were deeply anesthetized with a mixture of ketamine (100 mg/kg)/xylazine (10 mg/kg). The brains were then perfused (10% formalin; Sigma, Germany), collected, and post-fixed in the same fixator. The brains of a few animals were collected for real-time polymerase chain reaction analysis (RT-PCR).

#### Physical dissector method for neuronal counting

**Figure 1:**
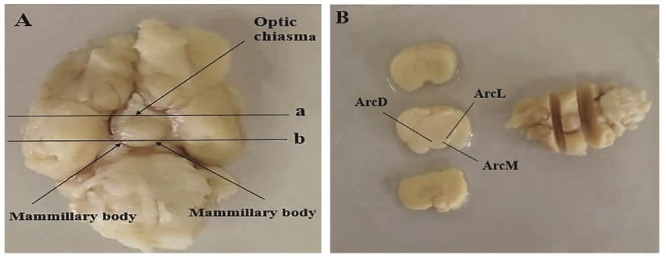
A) The inferior view of the rat brain. The anatomical location of the ARC is between the optic chiasm (a) and mammillary body (b). B) The brain's slice located between a and b in A was sampled histological preparation. ArcD: Dorsal arcuate nucleus, ArcL: Lateral arcuate nucleus, ArcM: Medial arcuate nucleus (based on Watson-Paxinus atlas).

The stereology was used to quantify ARC neurons' numerical density (Nv). For this purpose, a slice of the brain located between the optic chiasm and the mammary body was used (Figure 1). The tissue was blocked with paraffin (Merk, Germany) and then cut randomly at a thickness of 7 μ m using a microtome (Leitz 1512, Austria). According to the systematic random sampling method, at least 3 pairs of sections were taken, and several fields from each section were selected and imaged by a microscope (Olympus Bx51, Japan) equipped with a camera (Olympus DP71). More than 15 images were recorded for each of the brains. The images were placed under a counting frame without any direction. This method involved 2 consecutive cuts: reference and Look-up. The following formula was used to estimate Nv: 


$$Nv=\frac{\sum Q}{\sum \text{Frame}\times V}$$

$\sum {\;}_{Q}$ : number of neurons counted in all counting frames, $\sum {\;}_{\text{Frame}}$ : the number of counting frames, V: sampling volume

V = a ${\;}_{\text{ref}}$ × h

a ${\;}_{\text{ref}}$ : area of the sampling frame, h: depth of the sampling volume (distance between the upper surface of the first cut and the upper surface of the second cut, that is, if 2 consecutive cuts were used, it would be equal to the thickness of the cut).

### RT-PCR examination

Since perfusion destroys tissue RNA, brains are immediately removed from the skull without perfusion, and a coronal section was made in front of the optic chiasm and behind the mamillary bodies, as shown in figure 1. The slices were placed in the RNA shield solution (DENA Zist, Iran) and transferred to a -80 C freezer to protect the tissue RNA before the RNA extraction stage. A specific primer for the genes was designed (Table II).

For extracting the RNA, the powdered tissue was poured into a microtube in liquid nitrogen. After vortexing and adding the required materials according to the instructions of the kit (DENA Zist, Iran) at specific times and performing centrifugation, RNA was finally extracted. Next, cDNA was synthesized using the kit's instructions (Pars Tous, Iran) and after synthesizing template cDNA, polymerase chain reaction steps were performed, and the relative expression of genes was calculated using the RT-PCR method. Finally, the data was analyzed.

**Table 1:** Grouping of rats based on frequency and time interval of local PRP injection

**Groups^¥^**	**Stereotaxic surgery†**	**PRP injection††**			
		**Immediately**	**24 hr**	**48 hr**	**72 hr**
**Control**	—	—	—	—	—
**ARC ${\;}^{-}$ **		—	—	—	—
**PRP_imm_**			—	—	—
**PRP_24_**		—		—	—
**PRP_48_**		—	—		—
**PRP_72_**		—	—	—	
**PRP_imm.,24_**				—	—
**PRP_imm.,24.48_**					—
**PRP_imm.,24.48.72_**					
^¥^The results of the pilot tests indicated that the estrus cycle was not affected by stereotaxic cannulation of the ARC. Therefore, to reduce the number of animals and costs, the sham group was omitted from the main experiment. †Injection of 500 nmol/2 μ l QA into the hypothalamic ARC nucleolus. ††The injection times indicate the post-QA injection times. The times and injection frequencies were chosen to investigate the effects of these variables (the timing and frequency of PRP application) on each of the experimental groups. ARC^-^: Negative control group, PRP ${\;}_{\text{imm}}$ : Experimental group received PRP immediately after surgery. ARC: Arcuate nucleus, PRP: Platelet-rich plasma					

**Table 2:** Primers used for amplification with RT-PCR

**Gene**	**Primer sequences**
*Kiss1*	Forward: 5 ' -GATCTGCCTCTTCCAGAATG-3 ' Reverse: 5 ' -CAGGCATTAACGAGTTCCT-3 '
*Dynorphine*	Forward: 5 ' -CGTTCTCCATGTCTCCAAATA-3 Reverse: 5 ' -CCATCTCCTTAACCTACCAATC- 3 '
*Neurokinin B*	Forward: 5 ' -GTGGGACTTATGGGCAAG-3 ' Reverse: 5 ' -CCCTGTCTTTATGATGCAGT-3 '
* -actin*	Forward: 5 ' -CCTTCCTCCCAGAATGATCTC-3 ' Reverse: 5 ' -GATCCAGGCTTCACTTTTGC-3 '
RT-PCR: Real-time polymerase chain reaction,* Kiss1*: Kisspeptin-1, * β -actin*: Beta-actin	

### Ethical Considerations

All stages of this study were done according to international ethical codes for animal care and according to the Iran Biomedical Ethical Committee of Ferdowsi University of Mashhad; Mashhad, Iran (Code: IR.UM.REC.1401.213). The reason for using 90 rats in this experiment is as follows: the study involved examining control and 8 experimental groups, and bilateral lesioning of the hypothalamic ARC using stereotaxic surgery requires bilateral cannulation on both sides of the superior sagittal sinus. Due to the high mortality rate associated with cannula insertion, 10 rats were allocated to each group.

### Statistical Analysis

This experiment was conducted on 9 groups, each consisting of 10 female Wistar rats. Statistical analysis was carried out using GraphPad Prism 9 software (San Diego, California, USA) and SPSS version 24.0 (SPSS Inc., Chicago, Illinois, USA). A one-way ANOVA test was used to compare the experimental groups, followed by Tukey's post hoc test. The results were reported as mean ± standard deviation, and values of p < 0.05 were considered statistically significant.

## 3. Results

### Irregularity of estrous cycle

**Figure 2:**
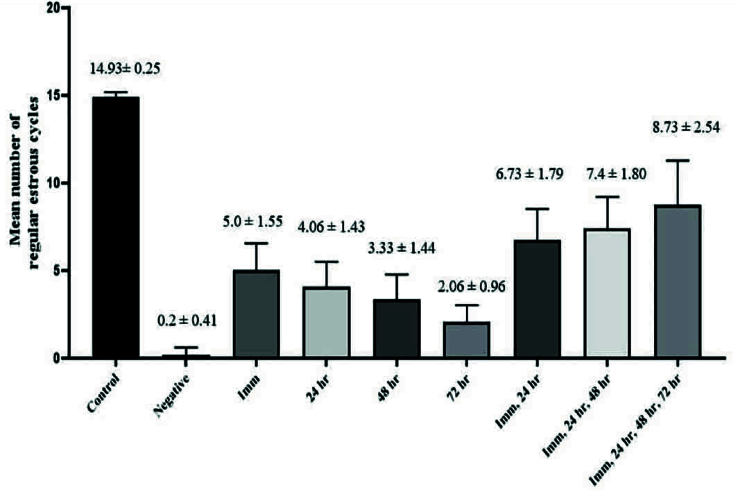
The average number of regular estrous cycles in control and experimental groups over 75 days.

As shown in figure 2, analysis of regular estrous cycle counts revealed a significant reduction in the number of regular estrous cycles in the QA treatment (negative group) compared to the control group (p < 0.001). Furthermore, treatment with PRP significantly increased the number of regular estrous cycles compared to the negative control group (p = 0.016). The closer the timing of PRP injection to the ARC nucleus destruction time, the more regular the cycles, which may indicate treatment efficacy. Also, groups receiving more than one time PRP injection showed more regular cycles compared to one injection (p = 0.035).

### The Nv of ARC neurons

**Figure 3:**
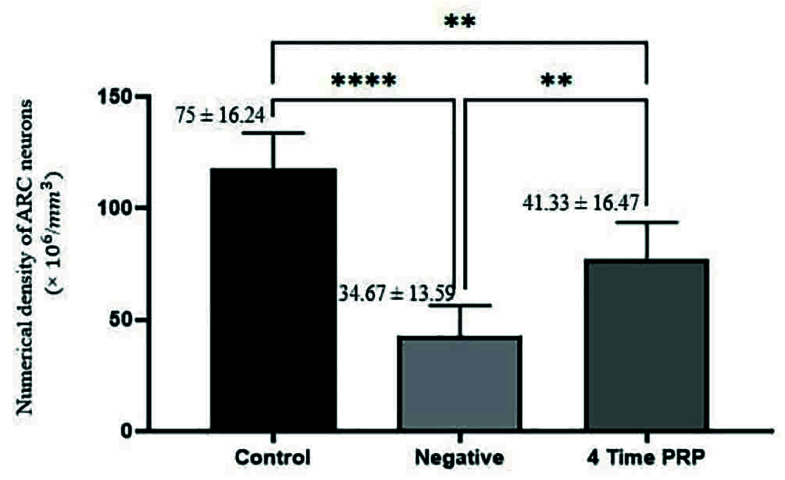
The Mean ± SD of the Nv of ARC neurons across control, negative control, and 4 PRP injection groups.The injection of PRP significantly increased Nv in the hypothalamic ARC. The results showed a significant difference between the control group and the negative control group (****P < 0.0001), indicating the negative effects of ARC damage on neuronal density. The group that received 4 PRP injections showed a significant increase in neuronal density compared to the negative control group (**P = 0.012).

The results of the comparison of Nv of ARC neurons are illustrated in figure 3.

### Results of histological study

**Figure 4:**
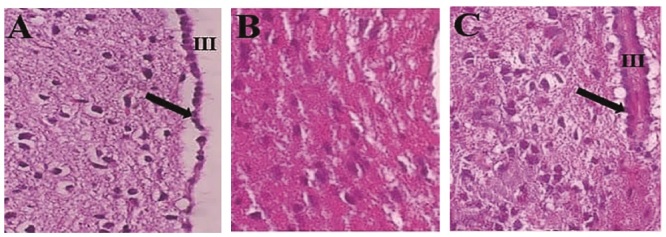
Histological appearance of the arcuate nucleus in A) Control, B) Negative control, and C) 4 times PRP injected groups. H&E staining, (X_40_), III, indicates the third ventricle, the arrow denotes the ependymal cells.

Figure 4 illustrates the micrographs of ARC section in control, negative control (ARC ${\;}^{-}$ ), and 4 times PRP injected groups. In comparison with control section, the negative control section seems to have most neurons degenerated; however, in the group that received 4 times PRP, the appearance of neurons is similar to control.

### Results of the RT-PCR analysis of KKNDY neurons in the ARC nucleus

**Figure 5:**
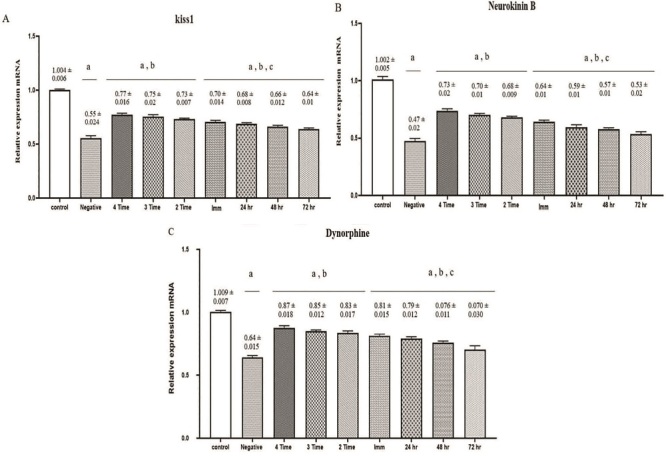
A, B, and C are the relative expression of Kiss1, NKB and Dyn mRNA in the ARC nucleus, respectively. A) Compared with control, B) Compared with negative control, C) Compared of multiple -PRP injections with one injection. Kiss1: Kisspeptin-1, NKB: Neurokinin B, Dyn: Dynorphin, ARC: Arcuate nucleus.

As illustrated in figure 5, the expression levels of all 3 genes, *KISS1, NKB,* and *DYN* were significantly elevated in the experimental groups compared to the negative control group (p < 0.001). Furthermore, a statistically significant difference was observed between the PRP-treated group and negative control group (p = 0.017). A notable variation was also found between the groups that received a single PRP injection (24, 48, and 72 hr) and those that underwent multiple injections, with the latter showing marginally higher expression levels (p = 0.028).

## 4. Discussion

The present study showed that QA-induced damage to the ARC of female rats reduces the number of regular estrus cycles. It was also observed that the local injection of PRP into the damaged ARC increases the number of regular estrus cycles. Furthermore, PRP application significantly increased the N ${\;}_{\text{V}}$ of ARC cells in negative control and 4 PRP injected groups, respectively. Additionally, the results of *KISS1, NKB*, and *Dyn* gene expression (Figure 5) revealed that an increase in N ${\;}_{\text{V}}$ of ARC cells is more likely due to the increase in KNDy neurons. Also, the effects of frequent PRP application are more evident than those of a single PRP injection. The protocol of post-injury PRP injection time and frequency of PRP application are considered in this context. The above observations indicate PRP's reconstructive/protective effects in the damaged ARC, especially when PRP was injected more than once.

Literature reviews show that the history of the use of PRP as a reparative substance for central nervous system damage dates back to the last 2 decades. Although the number of research records using PRP in repairing peripheral nervous system lesions is reasonable, the research records related to central nervous system damage are few. PRP is rich in growth factors and cytokines (22). Cytokines act as antioxidants and anti-inflammatory agents (23), and as our study showed, PRP counteracts QA's harmful effects and aids ARC healing. Alternatively, the growth factors in PRP are effective for tissue repair and regeneration (24, 25) likely to repair KNDy neurons (Figure 5). Studies focused on the effect of PRP on spinal cord repair demonstrated that it has a positive impact on the regeneration of damaged neurons (26); thus, the closer the time of using PRP to the time of injury, the more impressive its protective or therapeutic effects (27). PRP activates microglia and astrocytes, induces the expression of platelet-derived growth factor-B and intercellular adhesion molecule-1, promotes angiogenesis (28), enhances axonal growth through mechanisms associated with insulin-like growth factor-1, vascular endothelial growth factor, and significantly increases myelin thickness (29). Previous studies have demonstrated that after binding to their receptors (30), the growth factors in PRP elevate the expression of the anti-apoptotic protein BCL-2, consequently promoting cell survival and proliferation (22, 31). This data is in line with our results indicating an increased neuronal population of damaged ARC after PRP treatment. PRP injection significantly (p = 0.012) increased neuronal density in the damaged ARC, with a significant difference observed between the healthy control and negative control groups, confirming the detrimental effects of ARC injury. The group that received 4 PRP injections showed a notable improvement in neuronal density compared to the negative control, supporting the role of PRP in promoting neuronal regeneration. PRP enhances the proliferation and secretion of neurotrophic factors by glial cells under laboratory conditions (32).

Studies examining the principal growth factors found in PRP and their role in nerve repair demonstrated that platelet-derived growth factors enhance proliferation, differentiation, and migration (33) and therefore, contribute to cell survival and antiapoptotic processes (34), angiogenesis, and induction of cytokine secretion (35). These growth factors are instrumental in, multiple facets of, wound healing and tissue regeneration processes (36). Their collective actions orchestrate the intricate mechanisms involved in repairing damaged tissues and restoring normal function (37). Our cytological results indicated the normalization of estrus cycles after PRP treatments, which may be considered for repairing the damaged ARC and probable effects of growth factors and cytokines and their antiapoptotic effects.

This study examined the expression levels of *KISS, NKB*, and *DYN* genes in KNDy neurons using RT-PCR, revealing a significant increase in the PRP-treated groups. Kiss1, a key regulator of the hypothalamic-pituitary-gonadal axis, showed a substantial rise, indicating that PRP enhances reproductive functions by boosting the secretion of GnRH. NKB also increased, confirming its role in GnRH release. Interestingly, Dyn, which typically acts as an inhibitor, showed an increase, which may represent compensation in response to KISS and NKB elevations. Multiple PRP injections had a greater effect on gene expression compared to single doses. Overall, these findings demonstrate the potential of PRP in modulating reproduction regularity.

Due to the biological differences between humans and rats, further studies and clinical trials on humans are necessary for confirmation of PRP effects on recovery of human menstrual cycles. Although the 2.5-month study period from certain point of views may not be sufficient for a complete evaluation of tissue regeneration and the long-term effects of PRP. Also, because the central nervous system regenerates slowly, long-term studies could provide more information on the stability of ARC regeneration as well as regulation and restoration of the estrous cycle.

## 5. Conclusion

In conclusion, an increase in the number of regular estrous cycles (Figure 2), a significant increase in ARC N ${\;}_{\text{V}}$ (Figure 3), as well as a relative increase in Kisspeptin, NKB, and Dyn mRNA in the ARC nucleus (Figure 5) in rats treated by PRP indicate to the protective/regenerative effect of PRP. These findings underscore the therapeutic efficacy of PRP in attenuating neurodegenerative conditions. Employing PRP as a clinical approach for repairing damage to nervous systems holds significance, and ongoing research in this field has the potential to advance further medical and therapeutic benefits.

Overall, the results of the present study showed that (1) ARC is an essential part of hypothalamic-pituitary-gonadal axis, (2) the potential effect of PRP in repairing the damaged ARC, and (3) introduced an animal model for examining the reconstructive effects of natural or the chemical agents in central nervous system with overt (estrus cycle) outcome.

##  Data Availability

The supporting data of this study are available from the corresponding author upon reasonable request.

##  Author Contributions

M. Behnam Rassouli, A. Moghimi, Z. Neshati, and E. Abbasi designed the study and conducted the research. M. Behnam Rassouli, A. Moghimi, Z. Neshati, and E. Abbasi monitored, evaluated, and analyzed the results of the study. Further, M. Behnam Rassouli, A. Moghimi, and E. Abbasi reviewed the article. All authors approved the final manuscript and take responsibility for the integrity of the data.

##  Conflict of Interest

The authors declare that there is no conflict of interest.
